# Effect of grass pollen immunotherapy on clinical and local immune response to nasal allergen challenge

**DOI:** 10.1111/all.12608

**Published:** 2015-04-06

**Authors:** G. W. Scadding, A. O. Eifan, M. Lao‐Araya, M. Penagos, S. Y. Poon, E. Steveling, R. Yan, A. Switzer, D. Phippard, A. Togias, M. H. Shamji, S. R. Durham

**Affiliations:** ^1^ Allergy and Clinical ImmunologyNational Heart and Lung InstituteImperial College LondonLondonUK; ^2^ Immune Tolerance NetworkBethesdaMDUSA; ^3^ The National Institute of Allergy and Infectious DiseasesBethesdaMDUSA

**Keywords:** allergic rhinitis, chemokine, cytokine, Th2, tryptase

## Abstract

**Rationale:**

Nasal allergen provocations may be useful in investigating the pathophysiology of allergic rhinitis and effects of treatments.

**Objective:**

To use grass pollen nasal allergen challenge (NAC) to investigate the effects of allergen immunotherapy in a cross‐sectional study.

**Methods:**

We studied nasal and cutaneous responses in untreated subjects with seasonal grass‐pollen allergic rhinitis (*n* = 14) compared with immunotherapy‐treated allergics (*n* = 14), plus a nonatopic control group (*n* = 14). Volunteers underwent a standardized NAC with 2000 biological units of *timothy grass* allergen (equivalent to 1.3 μg major allergen, Phl p5). Nasal fluid was collected and analysed by ImmunoCAP and multiplex assays. Clinical response was assessed by symptom scores and peak nasal inspiratory flow (PNIF). Cutaneous response was measured by intradermal allergen injection. Retrospective seasonal symptom questionnaires were also completed.

**Results:**

Immunotherapy‐treated patients had lower symptom scores (*P* = 0.04) and higher PNIF (*P* = 0.02) after challenge than untreated allergics. They had reduced early (*P* = 0.0007) and late (*P* < 0.0001) skin responses, and lower retrospective seasonal symptom scores (*P* < 0.0001). Compared to untreated allergics, immunotherapy‐treated patients had reduced nasal fluid concentrations of IL‐4, IL‐9 and eotaxin (all *P* < 0.05, 8 h level and/or area under the curve comparison), and trends for reduced IL‐13 (*P* = 0.07, area under the curve) and early‐phase tryptase levels (*P* = 0.06).

**Conclusions:**

Nasal allergen challenge is sensitive in the detection of clinical and biological effects of allergen immunotherapy and may be a useful surrogate marker of treatment efficacy in future studies.

Allergic rhinitis is common, troublesome, costly and associated with asthma 1, 2, 3. Specific allergen immunotherapy is an effective treatment 4, particularly for seasonal allergic rhinitis. Clinical trials of allergen immunotherapy face several challenges, including standardization of allergen exposure between individuals, seasons and locations. Additionally, the primary outcome – combined symptom and medication score – may be subject to poor compliance, the result being that large numbers of participants are typically required.

We have previously described nasal challenges with grass pollen, accompanied by collection and analysis of mediators in nasal fluid 5. Response to nasal challenge may serve as a surrogate for seasonal symptoms 6, allowing assessment outside of pollen seasons, control of doses, and real‐time recording of symptoms. As such, nasal challenges and, more recently, environmental exposure chambers have been used to assess responses to allergen immunotherapy 7, 8. The effects of allergen immunotherapy on mediators in nasal fluid have also been investigated, with regard to ragweed 9, cat dander 7, grass pollen 10 and silver birch pollen 11, demonstrating suppression of histamine 9, kinins 12, tryptase, eosinophil cationic protein (ECP) 10 and IL‐5 11. Combining clinical and immunological outcomes has the benefit of providing insight into the mechanisms of allergic inflammation and immunotherapy.

We aimed to elaborate on this approach, investigating clinical outcomes of nasal challenge, their biological correlates, and relationship with seasonal symptoms. We hypothesized that patients receiving grass pollen immunotherapy would show blunted clinical and immunological responses to nasal challenge compared with untreated grass pollen allergics. We describe the results of a pilot, proof‐of‐concept, cross‐sectional study.

## Methods

### Participants

Volunteers were recruited from the allergy clinic at the Royal Brompton Hospital and from a database of previous study volunteers. Immunotherapy‐treated volunteers had a history of grass‐pollen seasonal allergic rhinitis for at least 2 years, positive skin prick (>3 mm wheal) and specific IgE (>0.70 IU/ml) to timothy grass extract and were receiving subcutaneous or sublingual timothy grass pollen allergen immunotherapy (Aquagen SQ, *Phleum pratense*, 100 000 SQ‐U/ml or Grazax 75 000 SQ‐U; both from ALK‐Abello, Hørsholm, Denmark). Untreated allergics had the same history of symptoms and positive skin and specific IgE tests, but no history of treatment with allergen immunotherapy. Nonatopic controls had no history of allergic disease, negative skin prick tests to timothy grass and other common aeroallergens, and negative specific IgE. Exclusion criteria were perennial rhinitis, chronic or recurrent sinusitis, current smoking or >5 pack‐year history, perennial asthma, and FEV1 <70% predicted at screening. Participants had not used nasal corticosteroids or other anti‐allergy medications for at least 2 weeks prior to assessment. The study was approved by the National Research Ethics Service, London – Camberwell St Giles office, and by the Research Office of the Royal Brompton and Harefield NHS Foundation Trust. Written, informed consent was obtained before study procedures were carried out.

### Study design

The study consisted of two visits: a screening visit including spirometry, skin prick testing to 12 common aeroallergens, ImmunoCAP‐specific IgE to timothy grass major allergen Phl p5 and total IgE (Phadia/Thermo Scientific, Uppsala, Sweden); then, for suitable candidates, a second visit for grass pollen nasal challenge. Challenge visits were conducted between February and April 2013, outside of the grass pollen season. Examiners were blind to the status of the participants. On the day of the challenge, volunteers recorded their baseline nasal symptoms according to a verified scoring system: total nasal symptom score (TNSS) 13, a 12‐point scale with four categories: sneezing, nose running, nose blockage and itching, each rated from 0 to 3. Additionally, the best of three measures using a Youlten nasal peak flow meter was recorded. Immediately afterwards, absorptive polyurethane sponges were placed into both nostrils to collect nasal fluid. Following this, participants underwent a nasal lavage (SinusRinse; NeilMed, Santa Rosa, CA, USA). The above procedures were repeated at 30 min after lavage. A further 10 min later, participants underwent a grass pollen nasal allergen challenge (NAC). TNSS, peak nasal inspiratory flow (PNIF) and nasal fluid were recorded/collected at 5, 15, 30 and 60 min after challenge, then at hourly intervals to 8 h. Between 1 and 2 h after nasal challenge, participants underwent an intradermal injection of 1 BU (biological unit; equivalent to 0.7 ng major allergen) of timothy grass extract on the outer surface of the forearm. Wheal response was recorded at 15 min, and late‐phase infiltration at 8 h, using a pencil‐friction technique described previously 14. Additionally, 15 min prior to nasal challenge, volunteers summarized the overall severity of their symptoms during the previous season (May–July 2012) on a retrospective 18‐point scale, with six categories: sneezing, nose running, nose blockage, nose itching, eye itching and eye watering/redness, each rated 0–3, giving a total score of 0–18.

### Nasal allergen challenge

Timothy grass pollen extract (Aquagen SQ; ALK‐Abello, Hørsholm, Denmark) was reconstituted at 100 000 SQ‐U/ml (equivalent to 30 000 BU/ml or 20.2 μg/ml major allergen) in albumin‐based diluent (ALK‐Abello), before 1 in 3 dilution in normal saline to a concentration of 33 333 SQ‐U/ml (10 000 BU/ml). Two hundred and thirty microlitres was then added to disposable Bi‐dose nasal applicator devices (Aptar Pharma, Louveciennes Cedex, France), manufactured to provide two 100‐μl sprays. Each participant received one 100‐μl spray of allergen to each nostril, applied by an examiner. Participants were asked not to sniff strongly or blow their nose in the first 5 min after allergen application.

### Collection and processing of nasal fluid

A 20 × 15 × 5 mm piece of synthetic polyurethane sponge (RG 27 grau; Gummi‐Welz GmbH & Co., Neu‐Ulm, Germany) was inserted by an examiner into each of the volunteer's nostrils, under direct vision using croc forceps and a Thuddicum's nasal speculum. Sponges were left in place for 2 min before removal and then added to 2‐ml centrifuge tubes with indwelling 0.22‐μm cellulose acetate filters (Costar Spin‐X; Corning, Corning, NY, USA). Tubes were kept briefly on ice before 100 μl of elution buffer [Milliplex Assay Buffer; Millipore, Darmstadt, Germany; PBS pH 7.4, BSA (1%), Tween‐20 (0.05%), sodium azide (0.05%)] was added on top of the sponge and the tube centrifuged at 4500 rcf at 4°C for 10 min. The isolated fluid was pipetted into Eppendorf tubes and stored at −80°C.

After thawing, nasal fluid was analysed for cytokines and chemokines using a human cytokine/chemokine magnetic bead panel 96‐well plate assay (Milliplex Map Kit; Millipore) and a Luminex xMAP Magpix platform (Millipore), according to the manufacturer's instructions. Samples of 25 μl of nasal fluid were analysed in duplicate alongside the manufacturer's standards and controls. Tryptase and ECP in nasal fluid were measured using an ImmunoCAP 100 machine (Phadia/Thermo Scientific) according to the manufacturer's instructions. Nasal fluid samples were diluted 1 in 5 in assay diluent (ImmunoCAP IgE/ECP/Tryptase Diluent; Thermo Scientific) and run alongside calibrators and curve controls.

### Statistical analysis

A commercial software package (GraphPad Prism version 5.04 GraphPad Software, Inc., La Jolla, CA, USA) was used. The prespecified primary outcome was the combined, equally weighted early (0–1 h)‐ and late (1–8 h)‐phase TNSS area under the curve (EPR AUC + LPR AUC/7), in immunotherapy‐treated volunteers *vs* untreated allergics. Secondary outcomes included the combined, equally weighted early‐ and late‐phase change from baseline PNIF (∆PNIF) AUC, and the separate early‐ and late‐phase AUCs for TNSS and ∆PNIF, as well as early‐ and late‐phase skin responses to intradermal allergen injection. Based on previous results 6, the primary comparisons for nasal fluid mediators were made at 8 h, with the exception of tryptase at 5 min; secondary analyses included AUC for the full 8 h. Within‐group comparisons were made by Wilcoxon's matched‐pairs test, between‐group comparisons by Mann–Whitney *U*‐test and correlations by Spearman's rank correlation coefficient. *P*‐values < 0.05 were considered statistically significant.

The study was powered based on the surface area of the late‐phase skin response to intradermal allergen injection. Previous studies 14, 15 have revealed up to 90% decrease after at least 3 months of immunotherapy compared to pretreatment response. We calculated that inclusion of 12 participants per group would provide 90% power to detect a 50% difference between groups. Other outcomes were exploratory.

## Results

### Participant demographics

Characteristics of the 14 immunotherapy‐treated volunteers, 14 untreated allergics and 14 nonatopic controls recruited are given in Table 1. Of the 14 immunotherapy patients, six were taking *Grazax* 75 000 SQ‐U sublingual tablets once daily, all for at least 6 months, and eight were receiving monthly injections of 100 000 SQ‐U *Aquagen SQ* timothy grass pollen extract, all having reached maintenance dose following a standard up‐dosing protocol (both from ALK‐Abello; see Table S1 in supporting information).

**Table 1:** Demographic characteristics of participants. Results presented as median (range)

	Nonatopics	Untreated allergics	Immunotherapy
*n* (M : F)	14 (5 : 9)	14 (9 : 5)	14 (6 : 8)
Age	35.5 (24 to 59)	31 (23 to 55)	38.5 (19 to 70)
Timothy grass IgE (kU/l)	0.01 (0.01 to 0.05)	9.7 (2.2 to 79.3)	28.2 (1.17 to >100)
Total IgE (kU_A_/l)	19.3 (<2 to 114)	138.5 (62.1 to 674)	188.5 (22.8 to 2305)
Mono : polysensitized	n.a.	7 : 7	7 : 7

### Clinical response to nasal allergen challenge

**Figure 1:**
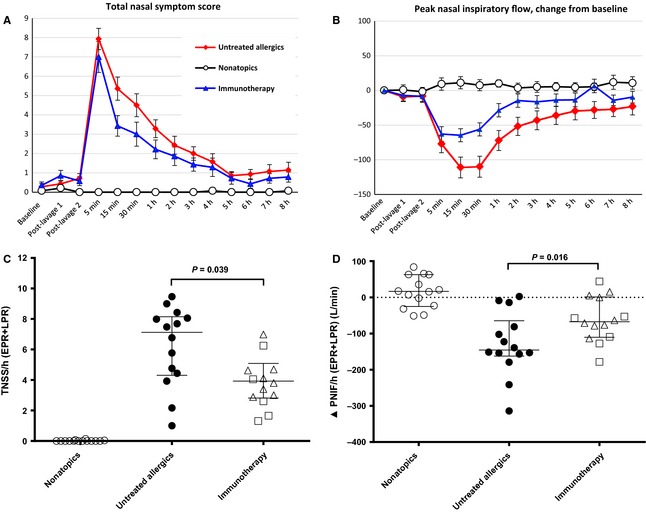
Response to nasal allergen challenge. A, total nasal symptom score (TNSS); B, change from baseline peak nasal inspiratory flow (∆PNIF); both mean ± SE. C, TNSS per hour combined early (EPR, 0–1 h)‐ and late (LPR, 1–8 h)‐phase responses with equal weighting; D, ∆PNIF per hour combined EPR and LPR; both median and interquartile range, between‐group comparisons by Mann–Whitney *U*‐test.

The immunotherapy group had reduced TNSS compared to untreated allergics (*P* = 0.039, Fig. 1), particularly during the early phase (*P* = 0.027, see Fig. S1 in supporting information). Immunotherapy patients had a smaller reduction in PNIF compared to untreated allergics (*P* = 0.016, Fig. 1) and, again, most pronounced in the early phase (*P* = 0.014, Fig. S1).

### Local nasal biomarkers

**Figure 2:**
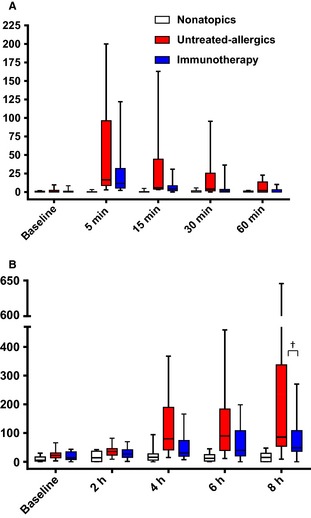
A, nasal fluid tryptase; B, nasal fluid eotaxin; both pg/ml, median and interquartile range. †*P* < 0.1, untreated allergics *vs* immunotherapy at 8 h, Mann–Whitney *U*‐test.

Nasal fluid tryptase levels peaked at 5 min postchallenge. The peak was blunted in immunotherapy patients – median 11.4 pg/ml *vs* 16.5 pg/ml in untreated allergics – but the difference did not reach statistical significance (*P* = 0.2; Fig. 2). Nonetheless, the levels remained reduced in immunotherapy patients at subsequent time points, narrowly missing statistical significance at 30 min (*P* = 0.06 *vs* untreated allergics; *P* = 0.1, AUC comparison, 0–60 min). Eotaxin levels were lower at 8 h in immunotherapy patients compared to untreated allergics, 48.8 pg/ml *vs* 86.0 pg/ml, with a trend to statistical significance (*p* = 0.08, Fig. 2 and Table S2); overall levels across the full 8 h were significantly lower (*P* = 0.04, AUC comparison).

**Figure 3:**
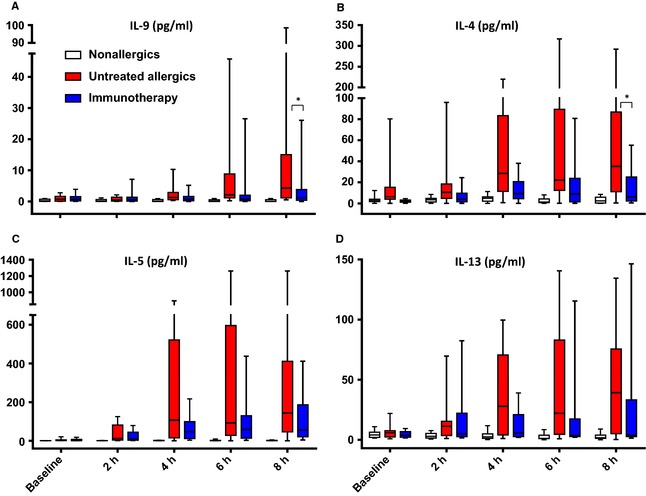
A, nasal fluid IL‐9; B, IL‐4; C, IL‐5; D, IL‐13; median and interquartile range. *P < 0.05, untreated allergics *vs* immunotherapy, 8 h, Mann–Whitney *U*‐test.

At 8 h, nasal fluid IL‐4 (*P* = 0.027) and IL‐9 (*P* = 0.049) were significantly reduced in immunotherapy patients compared to untreated allergics. The levels of IL‐5, IL‐8, IL‐10, IL‐13, MDC, RANTES and ECP were all lower at 8 h in immunotherapy patients (Fig. 3, Tables S2 and S3), without reaching statistical significance. AUC analysis for IL‐13 revealed a trend towards lower levels across the whole 8 h (*P* = 0.07).

### Cutaneous allergen response

**Figure 4:**
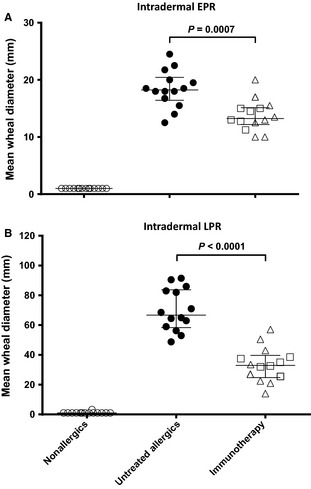
Response to intradermal injection of 1 BU purified timothy grass pollen allergen at 15 min (A, early‐phase response, EPR) and 8 h (B, late‐phase response, LPR). Mean of longest diameter and perpendicular diameter at midpoint of longest diameter, in millimetres. Median and interquartile range shown; between‐group comparisons by Mann–Whitney *U*‐test. Squares represent individuals receiving sublingual immunotherapy; and triangles, subcutaneous immunotherapy.

The early‐phase (15 min) cutaneous wheal response to 1 BU intradermal grass pollen allergen injection was smaller in immunotherapy patients than in untreated allergics (27% smaller, *P* < 0.0007), as was the late‐phase response (51% smaller, *P* < 0.0001 Fig. 4).

### Retrospective seasonal symptom scores and correlations

Overall retrospective seasonal symptom scores were significantly lower in the immunotherapy‐treated group than in the untreated allergics, median 6 *vs* 15 (*P* < 0.0001; Fig. S2).

In untreated allergics, ∆PNIF response to NAC correlated with seasonal symptom scores (early phase, *r* = −0.59, *P* = 0.021; combined early and late phase, *r* = −0.54, *P* = 0.036, Fig. S3). In the two allergic groups, overall seasonal symptom scores correlated with TNSS and PNIF responses to nasal challenge and with skin late‐phase intradermal allergen response (*r* = 0.62, *P* = 0.0004; *r* = −0.65, *P* = 0.0002; and *r* = 0.60, *P* = 0.0007, respectively, Fig. S4).

## Discussion

This study demonstrates the effects of grass pollen allergen immunotherapy on responses to NAC: reduced symptoms, improved PNIF, lower nasal fluid Th2 cytokines and chemokines, plus reduced early‐ and late‐phase cutaneous responses. These results provide insight into the mechanisms of specific allergen immunotherapy and suggest a possible role for NAC as a surrogate clinical outcome in future immunotherapy trials.

Researchers were blinded to the status of the participants in this study. PNIF, nasal fluid biomarkers and skin responses were objective measures and were concordant with symptom scores. Immunotherapy‐treated individuals had equal or greater baseline grass pollen sensitization than untreated allergics.

The cross‐sectional design has limitations – selection bias, recall bias, absence of baseline data – yet, as proof‐of‐concept, these data are valuable, being more detailed and complete than preceding studies. The inclusion of both SCIT and SLIT patients introduced heterogeneity, as did the variable treatment durations. Despite these limitations – which might be expected to reduce power – we were still able to detect significant differences between treated and untreated volunteers. The study is underpowered for comparison between SLIT and SCIT, but this was never the intention.

Investigators have described rapid release of tryptase 5, 16 and slower increase in Th2 cytokines/chemokines 5, 17, 18, 19, 20 after NAC. Similar profiles, albeit at lower magnitude, have been described in seasonal assessments, without direct provocation 21, 22, 23. Few studies have compared NAC with seasonal symptoms 6, although an allergen environmental exposure chamber has recently shown good correlation 24. The correlations we found between seasonal symptom scores and responses to NAC, though significant, were modest and insufficient to make predictions on an individual basis. Conversely, there is no correlation between specific IgE levels and response to nasal provocation, meaning provocation cannot be substituted by serum testing 25. No correlations were evident between biomarkers after NAC and seasonal symptoms (data not shown). It would have been ideal to include more detailed seasonal assessments, including collection of nasal fluid in season, to allow comparison of biomarkers after NAC with those during natural seasonal exposure.

Previously, grass pollen immunotherapy has inhibited seasonal eosinophil infiltration and reduced IL‐5 mRNA in nasal tissue 26 and also IL‐5 protein in nasal fluid 11; clinical improvement was accompanied by an increase in local IFN‐γ : IL‐5 mRNA ratio 27; and seasonal IL‐9 mRNA was reduced alongside c‐kit+ mast cells 28. The results presented here are therefore largely in keeping with the current literature, but extend findings to cover a broader range of mediators in the same cohort, using noninvasive techniques.

Immunotherapy had a predominant effect on symptoms within the first hour after provocation. This effect on early responses has been described previously following ragweed immunotherapy 9. This might reflect greater biological suppression of early‐ than late‐phase mechanisms, but we saw suppression of mediators in both phases. Conversely, the TNSS clearly has maximum sensitivity during the early phase, and distinct late‐phase clinical responses to NAC are only present in a minority 5, 12. This is in contrast to bronchial allergen provocation where late responses are easily detected by falls in FEV1 and inhibited by allergen immunotherapy 29. Notably, the opposite pattern was seen in cutaneous allergen response, with a greater suppression of late rather than early phase, as described previously 12.

Concerning effects of immunotherapy, there was a late (4–8 h) suppression of Th2 cytokines, the strongest effects being apparent with IL‐4, IL‐9 and eotaxin. Whilst several researchers have demonstrated increases in peripheral blood T‐cell secretion of IL‐10 *in vitro* following immunotherapy, few have looked at local effects. In fact, the local IL‐10 response to allergen exposure is far from clear: Pilette et al. 30 found fewer IL‐10 mRNA+ cells in the nasal mucosa of allergics after NAC, whereas Benson et al. 21 reported increased levels of IL‐10 in nasal lavage in seasonal allergic rhinitis. We could not detect a clear IL‐10 response to nasal challenge in nasal fluid nor an effect of immunotherapy. Whilst ECP levels were elevated in allergics, we did not identify a significant treatment effect.

Nasal allergen provocation is convenient, allowing flexibility in comparison with seasonal assessments. There is also likely to be lower risk of incomplete data recording and loss to follow‐up. We have identified several potential local biomarkers of response to allergen immunotherapy – their utility needs to be borne out in larger, prospective studies. Additionally, these techniques should be extended to perennial allergens such as house dust mite where the relevance of allergic sensitization to symptoms is not always obvious.

In summary, this study demonstrates that grass pollen immunotherapy improves symptoms and peak nasal flow after allergen challenge, associated with reductions in early‐ and late‐phase local inflammatory mediators. Low reactivity to nasal provocation is associated with lower seasonal symptoms – hence, the model described here may be applicable as a surrogate end point for studies of allergen immunotherapy for seasonal allergic rhinitis.

## Funding

This research was funded by an Imperial College – Wellcome Trust Clinical PhD Fellow Programme, awarded to Guy W. Scadding (reference: 097881/Z/11/Z), and by a research grant from ALK‐Abello, Denmark. Stephen R. Durham and Mohamed H. Shamji were part‐funded by the Immune Tolerance Network (NIH Contract #N01 AI15416), an international clinical research consortium headquartered at the Benaroya Research Institute, Seattle, Washington, and supported by the National Institute of Allergy and Infectious Diseases, USA.

## Author contributions

Guy W. Scadding, Mohamed H. Shamji and Stephen R. Durham designed and developed the protocol for the study. Guy W. Scadding, Aarif Eifan and Martin Penagos were responsible for conducting the clinical study, assisted by Rachel Yan and Shun Y. Poon. Guy W. Scadding ran the laboratory assays, assisted by Amy Switzer and Mongkol Lao‐Araya. Guy W. Scadding performed the analysis of results, assisted by Shun Y. Poon and Esther Steveling. Deborah Phippard and Alkis Togias provided expertise concerning data interpretation. Guy W. Scadding wrote the manuscript, with final review by Stephen R. Durham.

## Competing interests

Stephen R. Durham has received payments for consultancies via Imperial College from ALK, Merck USA, Circassia, Stallergenes, Laboratorios Leti (manufacturers of allergy vaccines) and Juno Pharmaceuticals. He has received research funding via Imperial College London for investigator‐led projects from ALK, Biotech Tools, Merck USA, Stallergenes, Novartis, Laboratorios Leti and Regeneron Pharmaceuticals, funding from Boehringer Ingelheim to attend an international conference and an honorarium from Merck for giving a lecture unrelated to the current topic. The remaining authors have no competing interests to declare.

## Supporting information


**Figure S1.** Response to nasal allergen challenge. A, TNSS per hour for early‐phase response (EPR, 0–1 h, area under the curve). B, Change from baseline peak nasal inspiratory flow (∆PNIF) per hour for EPR. C, TNSS per hour for late‐phase response (LPR, 1–8 h). D, ∆PNIF per hour for LPR. Median ± IQR; comparisons by Mann–Whitney *U*‐test.
**Figure S2.** Overall seasonal symptom score (0–18, symptom‐free to maximal symptoms; 0–3, in each of six categories). Individual data points, median and interquartile range; comparisons by Mann–Whitney *U*‐test. Squares represent individuals receiving sublingual immunotherapy, triangles subcutaneous immunotherapy.
**Figure S3.** Correlations, by Spearman's rank correlation coefficient, between seasonal symptom scores and change from baseline peak nasal inspiratory flow (∆PNIF) in the first hour after challenge (A, EPR) and the equally weighted, combined early‐ and late‐phase responses (B, EPR + LPR), in untreated atopic volunteers. AUC, area under the curve.
**Figure S4.** Correlations, by Spearman's rank correlation coefficient, between seasonal symptom scores and response to nasal challenge. A, TNSS per hour combined, equally weighted, early‐ and late‐phase responses (EPR + LPR); B, change from baseline PNIF per hour combined, equally weighted, EPR + LPR; C, skin LPR to intradermal allergen injection. Nonatopic patients excluded from analysis.
**Table S1.** Characteristics of immunotherapy‐treated patients. Results given as median (range). SCIT, subcutaneous allergen immunotherapy; SLIT, sublingual allergen immunotherapy.
**Table S2.** Cytokines/chemokines and tryptase in nasal fluid; median (range). *P < 0.05, †*P* < 0.1, untreated allergics *vs* immunotherapy; all at 8 h by Mann–Whitney *U*‐test, except tryptase at 5 min.
**Table S3.** Cytokines/chemokines and ECP in nasal fluid; median (range).
