# Supplementary material for: Effect of grass pollen immunotherapy on clinical and local immune response to nasal allergen challenge
Source: Allergy. 2015 Apr 6;70(6):689–96. doi: 10.1111/all.12608 (PMC4826905; doi:10.1111/all.12608)
Supplement: Supplementary file 1 — Figure S1. Response to nasal allergen challenge. A, TNSS per hour for early‐phase response (EPR, 0–1 h, area under the curve). B, Change from baseline peak nasal inspiratory flow (∆PNIF) per hour for EPR. C, TNSS per hour for late‐phase response (LPR, 1–8 h). D, ∆PNIF per hour for LPR. Median ± IQR; comparisons by Mann–Whitney U‐test. Figure S2. Overall seasonal symptom score (0–18, symptom‐free to maximal symptoms; 0–3, in each of six categories). Individual data points, median and interquartile range; comparisons by Mann–Whitney U‐test. Squares represent individuals receiving sublingual immunotherapy, triangles subcutaneous immunotherapy. Figure S3. Correlations, by Spearman's rank correlation coefficient, between seasonal symptom scores and change from baseline peak nasal inspiratory flow (∆PNIF) in the first hour after challenge (A, EPR) and the equally weighted, combined early‐ and late‐phase responses (B, EPR + LPR), in untreated atopic volunteers. AUC, area under the curve. Figure S4. Correlations, by Spearman's rank correlation coefficient, between seasonal symptom scores and response to nasal challenge. A, TNSS per hour combined, equally weighted, early‐ and late‐phase responses (EPR + LPR); B, change from baseline PNIF per hour combined, equally weighted, EPR + LPR; C, skin LPR to intradermal allergen injection. Nonatopic patients excluded from analysis. Table S1. Characteristics of immunotherapy‐treated patients. Results given as median (range). SCIT, subcutaneous allergen immunotherapy; SLIT, sublingual allergen immunotherapy. Table S2. Cytokines/chemokines and tryptase in nasal fluid; median (range). *P < 0.05, †P < 0.1, untreated allergics vs immunotherapy; all at 8 h by Mann–Whitney U‐test, except tryptase at 5 min. Table S3. Cytokines/chemokines and ECP in nasal fluid; median (range). [file ALL-70-689-s001.pdf]

Type of immunotherapy	Treatment duration (months)
8 SCIT	6 (3 – 28)
6 SLIT	30.5 (7 – 60)

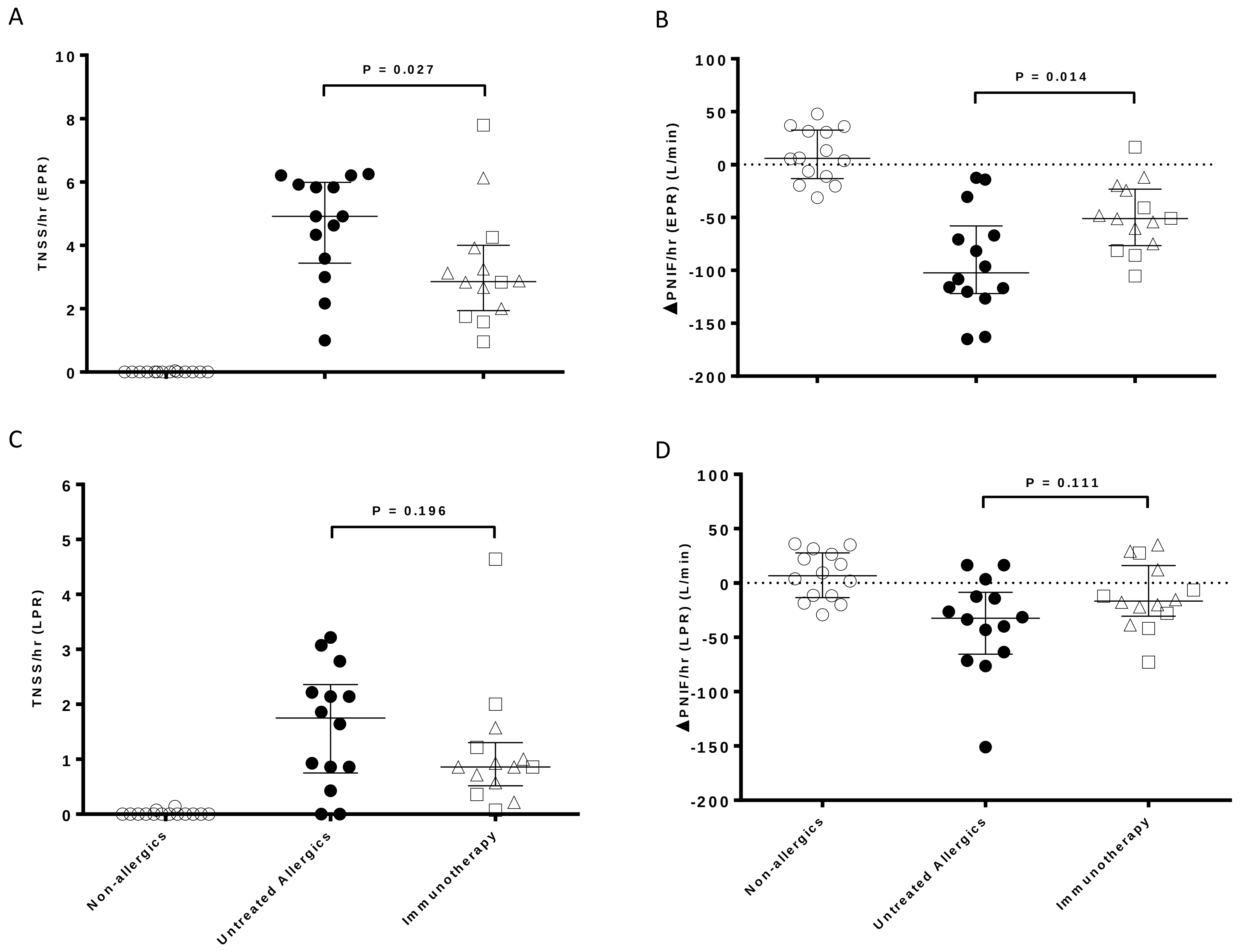
A
B
C
D
TNSS/hr (EPR)
ΔPNIF/hr (EPR) (L/min)
TNSS/hr (LPR)
ΔPNIF/hr (LPR) (L/min)
P = 0.027
P = 0.014
P = 0.196
P = 0.111
Non-allergics
Untreated Allergics
Immunotherapy

Mediator (pg/mL)	Time-point	Non-atopics	Untreated-allergics	Immunotherapy
tryptase	baseline	0.0 (0.0-2.0)	0.6 (0.0-9.7)	0.0 (0.0-8.5)
	5 minutes	0.0 (0.0-3.2)	16.5 (2.9-200.0)	11.4 (2.1-122.0)
	15 minutes	0.0 (0.0-4.8)	5.9 (3.1-163.0)	4.2 (0.0-30.9)
	30 minutes	0.0 (0.0-5.5)	3.9 (0.0-95.5)	1.4 (0.0-36.5)
	60 minutes	0.0 (0.0-2.3)	1.9 (0.0-22.7)	0.0 (0.0-10.3)
IL-4	baseline	2.9 (0.0-12.2)	6.3 (0.0-80.3)	2.3 (0.0-4.5)
	2 hours	3.5 (0.0-8.5)	10.5 (0.0-95.9)	4.1 (0.0-24.5)
	4 hours	4.9 (0.0-11.3)	28.5 (0.7-219.8)	9.3 (0.0-38.0)
	6 hours	1.3 (0.0-8.2)	22.1 (0.2-316.9)	9.1 (0.0-80.8)
	8 hours	2.4 (0.0-8.6)	35.1 (0.4-292.0)	6.3 (0.6-55.2)*
IL-5	baseline	0.9 (0.0-1.5)	2.1 (0.6-20.7)	1.8 (0.2-2.9)
	2 hours	0.9 (0.1-2.0)	12.4 (0.7-125.2)	9.0 (1.3-79.6)
	4 hours	1.2 (0.2-3.3)	106.2 (0.6-894.6)	46.9 (3.3-216.8)
	6 hours	1.2 (0.2-9.4)	93.0 (0.9-1262.8)	58.9 (2.3-436.9)
	8 hours	1.1 (0.0-4.9)	143.5 (0.9-1262.3)	55.9 (4.5-411.2)
IL-9	baseline	0.2 (0.0-0.9)	0.7 (0.0-2.8)	0.7 (0.0-3.9)
	2 hours	0.1 (0.0-1.2)	0.5 (0.0-2.1)	0.6 (0.0-7.1)
	4 hours	0.1 (0.0-0.9)	1.3 (0.3-10.3)	0.7 (0.0-5.2)
	6 hours	0.1 (0.0-1.0)	2.2 (0.2-45.8)	0.8 (0.0-26.6)
	8 hours	0.1 (0.0-1.0)	4.3 (0.5-98.5)	1.2 (0.0-26.1)*
IL-13	baseline	4.1 (1.4-11.0)	5.9 (1.0-22.0)	3.8 (1.5-9.4)
	2 hours	3.0 (0.7-7.7)	11.4 (1.2-69.7)	5.0 (1.6-82.5)
	4 hours	2.5 (0.2-11.9)	27.8 (1.1-99.5)	5.6 (2.0-39.0)
	6 hours	1.2 (0.0-8.5)	22.1 (1.0-141.0)	3.7 (2.2-115.5)
	8 hours	1.7 (0.0-9.0)	39.3 (0.3-134.4)	3.9 (1.4-146.4)
Eotaxin	baseline	5.5 (0.0-29.9)	22.0 (2.5-65.9)	13.3 (0.0-43.8)
	2 hours	13.9 (0.0-42.4)	35.7 (8.9-82.0)	26.9 (1.3-69.6)
	4 hours	15.5 (0.0-94.4)	80.2 (14.6-367.9)	31.2 (7.3-166.1)
	6 hours	11.9 (0.0-45.1)	89.9 (11.2-459.3)	39.9 (0.0-198.6)
	8 hours	14.9 (0.0-47.7)	86.0 (8.9-645.0)	48.8 (0.0-270.5)†

Mediator (pg/mL)	Time-point	Non-atopics	Untreated-allergics	Immunotherapy
IL-8	baseline	1009.9 (527.6-3333.5)	1384.4 (257.5-4168.5)	807.5 (71.0-2247.5)
	2 hours	655.1 (304.2-1702.5)	548.4 (250.0-1652.0)	872.8 (333.8-1575.1)
	4 hours	757.5 (419.8-2245.3)	1018.2 (313.4-2121.5)	867.3 (330.5-2417.1)
	6 hours	775.2 (164.4-3126.6)	1019.4 (313.5-3398.0)	1022.0 (346.9-4965.3)
	8 hours	965.0 (555.8-1903.3)	1540.9 (246.4-3851.3)	1126.6 (441.2-2738.4)
IL-10	baseline	2.3 (1.0-5.6)	4.2 (1.7-32.7)	3.0 (0.4-15.9)
	2 hours	6.1 (1.7-37.8)	8.7 (1.5-49.0)	1.0 (1.8-42.4)
	4 hours	5.3 (1.4-13.8)	17.6 (3.7-40.6)	7.7 (1.8-36.8)
	6 hours	5.4 (0.6-24.4)	16.0 (5.6-56.8)	6.0 (1.7-31.4)
	8 hours	5.9 (1.4-16.4)	18.4 (2.7-70.5)	8.5 (1.1-114.1)
MDC	baseline	50.9 (27.7-134.9)	98.2 (22.5-244.9)	68.2 (4.4-226.2)
	2 hours	69.7 (14.7-263.3)	105.2 (10.1-245.7)	98.3 (26.1-329.9)
	4 hours	63.4 (17.3-260.0)	75.1 (32.3-628.4)	86.5 (22.8-320.3)
	6 hours	49.7 (14.7-92.2)	70.0 (33.0-427.3)	71.2 (8.6-695.9)
	8 hours	39.9 (12.3-108.6)	127.5 (25.6-668.5)	109.1 (77.6-282.7)
RANTES	baseline	12.5 (6.0-131.2)	22.1 (5.6-73.5)	12.7 (1.3-68.7)
	2 hours	13.6 (3.8-205.0)	17.7 (5.3-94.1)	14.8 (5.3-129.6)
	4 hours	15.3 (3.8-123.9)	26.0 (8.5-52.7)	11.2 (4.4-62.5)
	6 hours	7.4 (2.8-139.8)	18.0 (6.2-72.5)	9.4 (3.0-79.3)
	8 hours	20.1 (1.3-175.6)	27.8 (2.9-41.0)	17.9 (2.5-129.4)
ECP	baseline	4.55 (3.2-16.3)	10.1 (4.9-14.7)	5.7 (3.4-14.3)
	2 hours	8.5 (4.8-13.7)	18.7 (11.7-45.5)	18.7 (9.4-91.3)
	4 hours	14.5 (7.0-36.9)	40.2 (23.0-96.1)	39.2 (25.2-134.4)
	6 hours	20.1 (4.4-25.8)	62.5 (25.9-75.8)	38.1 (17.7-79.2)
	8 hours	15.1 (8.9-21.2)	77.5 (41.4-96.7)	36.9 (20.4-81.2)

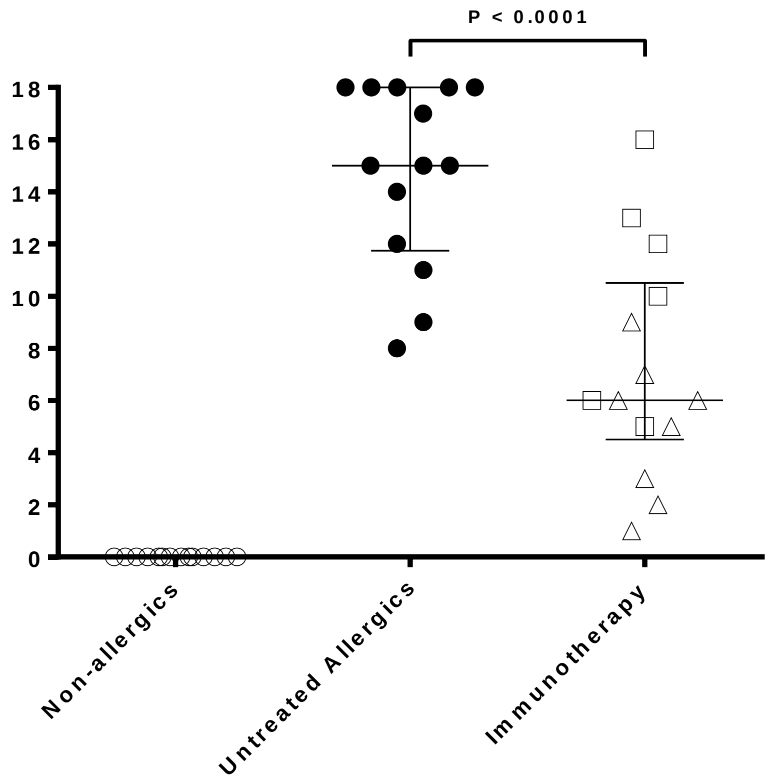
P < 0.0001
18
16
14
12
10
8
6
4
2
0
Non-allergics
Untreated Allergics
Immunotherapy

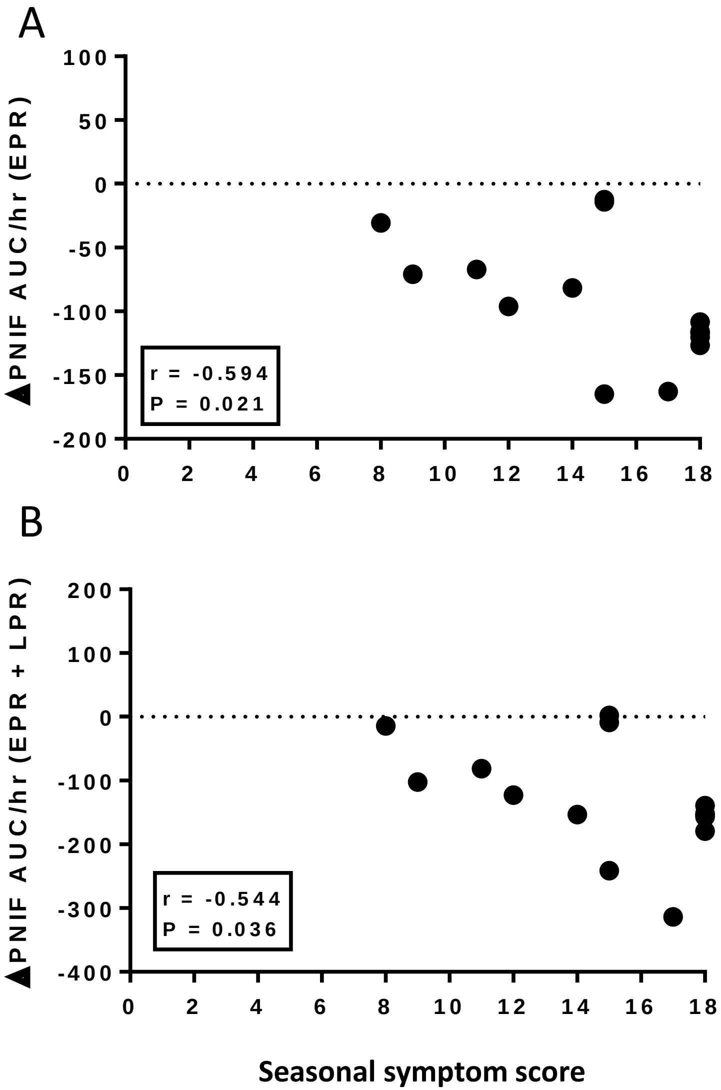
A
B
ΔPNIF AUC/hr (EPR)
ΔPNIF AUC/hr (EPR + LPR)
r = -0.594
P = 0.021
r = -0.544
P = 0.036
Seasonal symptom score

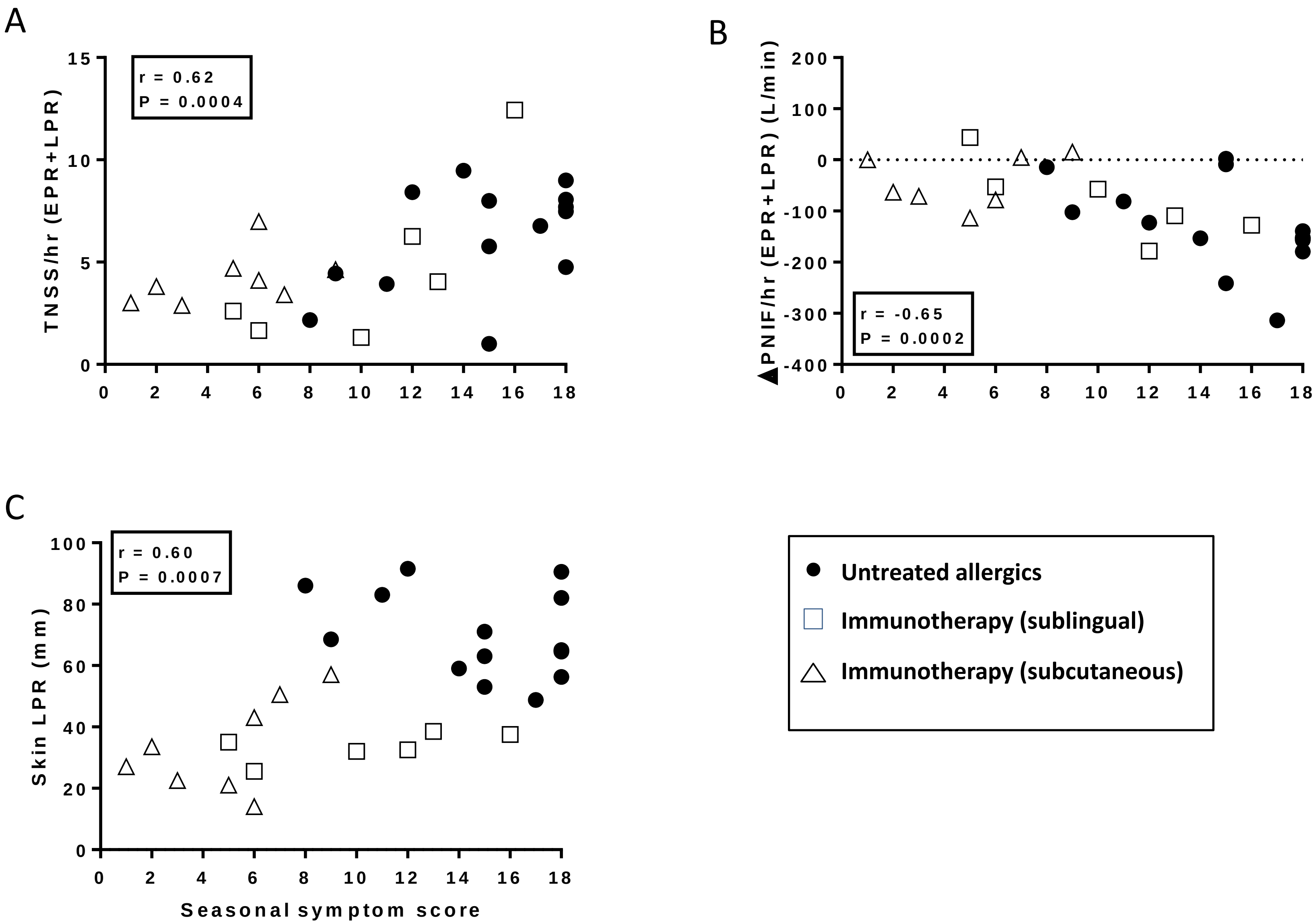

A
r = 0.62
P = 0.0004
TNSS/hr (EPR+LPR)
B
r = -0.65
P = 0.0002
ΔPNIF/hr (EPR+LPR) (L/min)
C
r = 0.60
P = 0.0007
Skin LPR (mm)
Seasonal symptom score
Untreated allergics
Immunotherapy (sublingual)
Immunotherapy (subcutaneous)
