# Supplementary material for: Effect of grass pollen immunotherapy on clinical and local immune response to nasal allergen challenge
Source: Allergy. 2015 Apr 6;70(6):689–96. doi: 10.1111/all.12608 (PMC4826905; doi:10.1111/all.12608)
Supplement: Supplementary file 2 [file ALL-70-689-s002.pptx]

## Slide 1
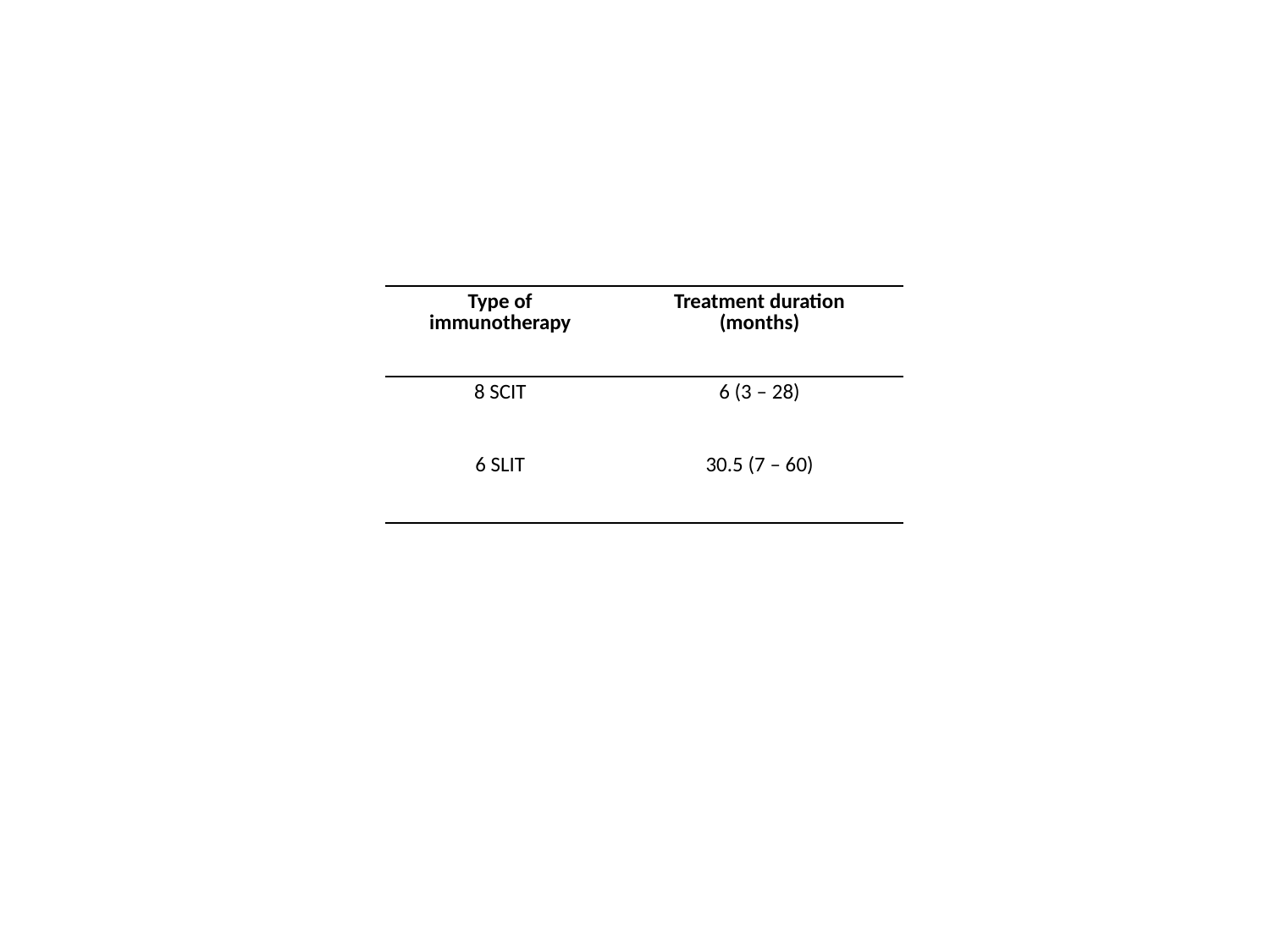

| Type of immunotherapy | Treatment duration (months) |
| --- | --- |
| 8 SCIT | 6 (3 – 28) |
| 6 SLIT | 30.5 (7 – 60) |

## Slide 2
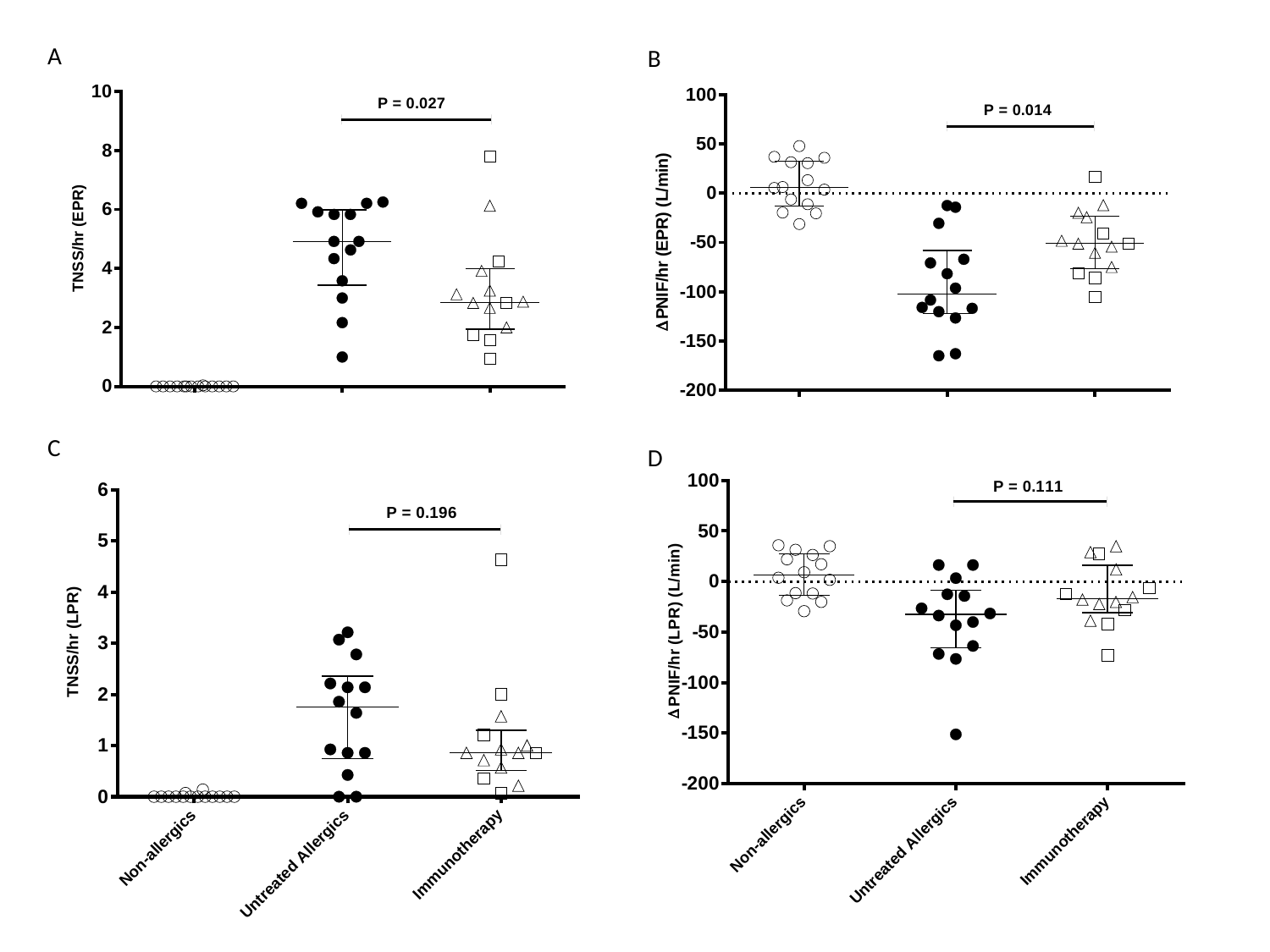

A
B
C
D

## Slide 3
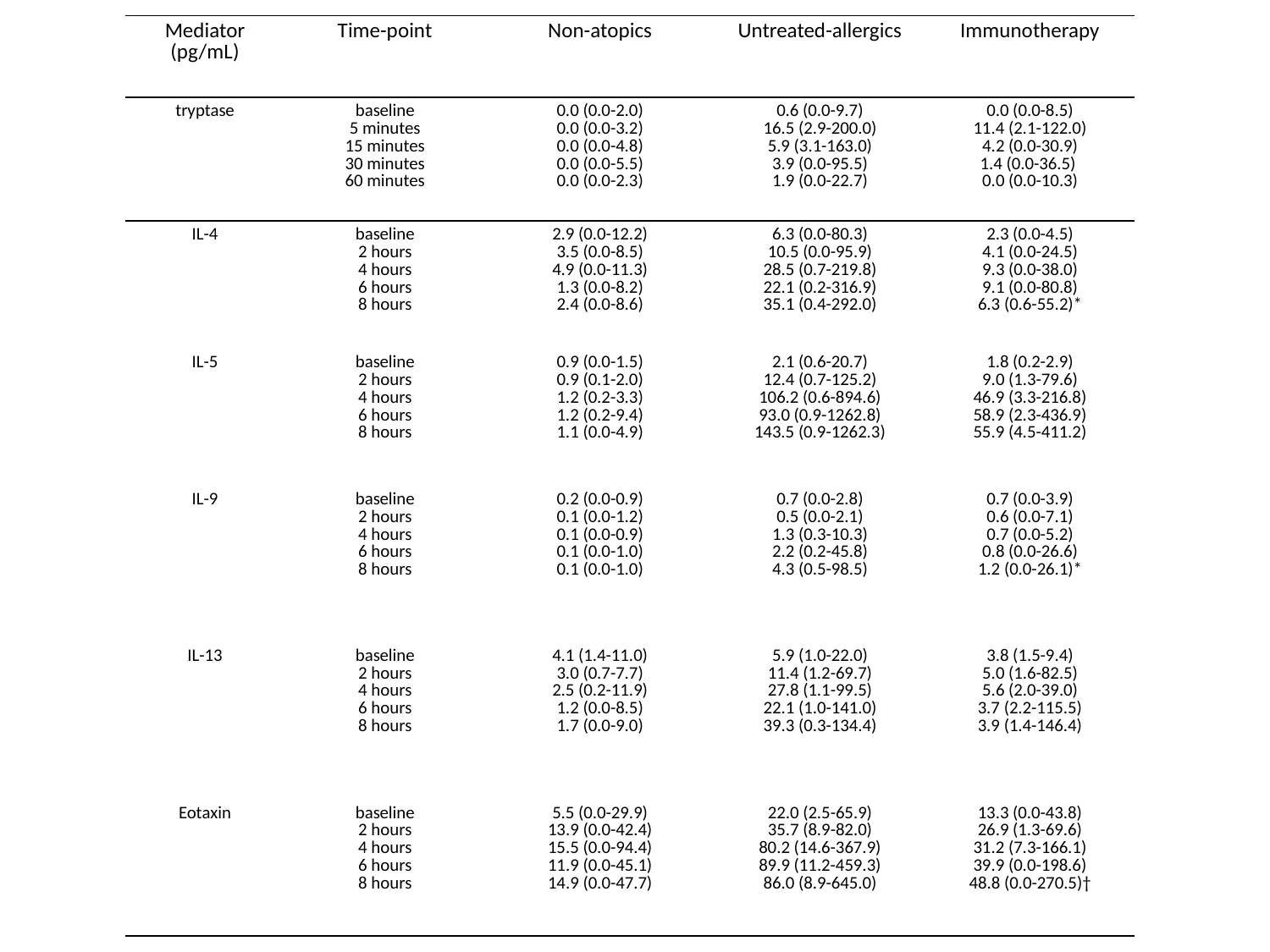

| Mediator (pg/mL) | Time-point | Non-atopics | Untreated-allergics | Immunotherapy |
| --- | --- | --- | --- | --- |
| tryptase | baseline 5 minutes 15 minutes 30 minutes 60 minutes | 0.0 (0.0-2.0) 0.0 (0.0-3.2) 0.0 (0.0-4.8) 0.0 (0.0-5.5) 0.0 (0.0-2.3) | 0.6 (0.0-9.7) 16.5 (2.9-200.0) 5.9 (3.1-163.0) 3.9 (0.0-95.5) 1.9 (0.0-22.7) | 0.0 (0.0-8.5) 11.4 (2.1-122.0) 4.2 (0.0-30.9) 1.4 (0.0-36.5) 0.0 (0.0-10.3) |
| IL-4 | baseline 2 hours 4 hours 6 hours 8 hours | 2.9 (0.0-12.2) 3.5 (0.0-8.5) 4.9 (0.0-11.3) 1.3 (0.0-8.2) 2.4 (0.0-8.6) | 6.3 (0.0-80.3) 10.5 (0.0-95.9) 28.5 (0.7-219.8) 22.1 (0.2-316.9) 35.1 (0.4-292.0) | 2.3 (0.0-4.5) 4.1 (0.0-24.5) 9.3 (0.0-38.0) 9.1 (0.0-80.8) 6.3 (0.6-55.2)\* |
| IL-5 | baseline 2 hours 4 hours 6 hours 8 hours | 0.9 (0.0-1.5) 0.9 (0.1-2.0) 1.2 (0.2-3.3) 1.2 (0.2-9.4) 1.1 (0.0-4.9) | 2.1 (0.6-20.7) 12.4 (0.7-125.2) 106.2 (0.6-894.6) 93.0 (0.9-1262.8) 143.5 (0.9-1262.3) | 1.8 (0.2-2.9) 9.0 (1.3-79.6) 46.9 (3.3-216.8) 58.9 (2.3-436.9) 55.9 (4.5-411.2) |
| IL-9 | baseline 2 hours 4 hours 6 hours 8 hours | 0.2 (0.0-0.9) 0.1 (0.0-1.2) 0.1 (0.0-0.9) 0.1 (0.0-1.0) 0.1 (0.0-1.0) | 0.7 (0.0-2.8) 0.5 (0.0-2.1) 1.3 (0.3-10.3) 2.2 (0.2-45.8) 4.3 (0.5-98.5) | 0.7 (0.0-3.9) 0.6 (0.0-7.1) 0.7 (0.0-5.2) 0.8 (0.0-26.6) 1.2 (0.0-26.1)\* |
| IL-13 | baseline 2 hours 4 hours 6 hours 8 hours | 4.1 (1.4-11.0) 3.0 (0.7-7.7) 2.5 (0.2-11.9) 1.2 (0.0-8.5) 1.7 (0.0-9.0) | 5.9 (1.0-22.0) 11.4 (1.2-69.7) 27.8 (1.1-99.5) 22.1 (1.0-141.0) 39.3 (0.3-134.4) | 3.8 (1.5-9.4) 5.0 (1.6-82.5) 5.6 (2.0-39.0) 3.7 (2.2-115.5) 3.9 (1.4-146.4) |
| Eotaxin | baseline 2 hours 4 hours 6 hours 8 hours | 5.5 (0.0-29.9) 13.9 (0.0-42.4) 15.5 (0.0-94.4) 11.9 (0.0-45.1) 14.9 (0.0-47.7) | 22.0 (2.5-65.9) 35.7 (8.9-82.0) 80.2 (14.6-367.9) 89.9 (11.2-459.3) 86.0 (8.9-645.0) | 13.3 (0.0-43.8) 26.9 (1.3-69.6) 31.2 (7.3-166.1) 39.9 (0.0-198.6) 48.8 (0.0-270.5)† |

## Slide 4
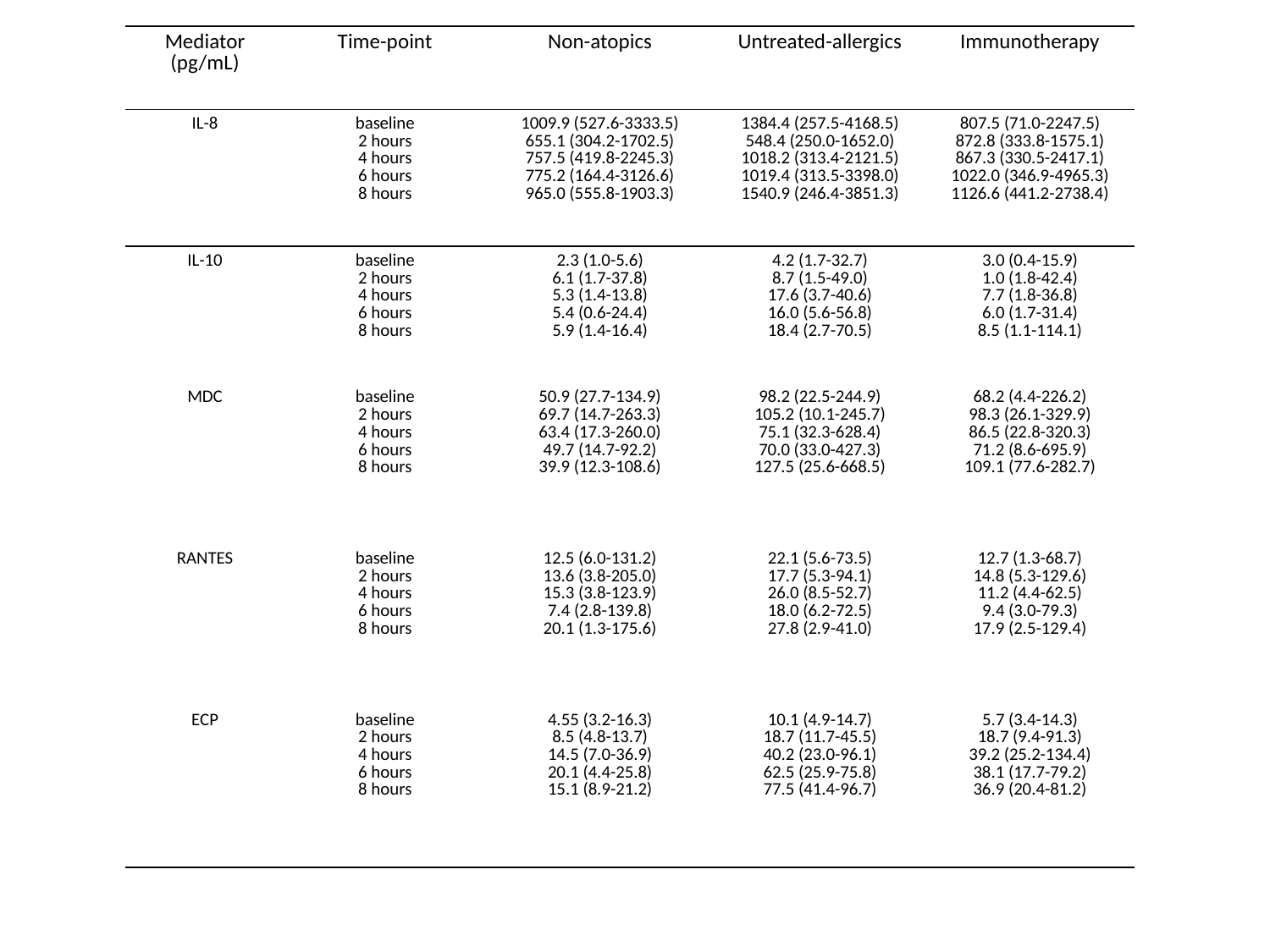

| Mediator (pg/mL) | Time-point | Non-atopics | Untreated-allergics | Immunotherapy |
| --- | --- | --- | --- | --- |
| IL-8 | baseline 2 hours 4 hours 6 hours 8 hours | 1009.9 (527.6-3333.5) 655.1 (304.2-1702.5) 757.5 (419.8-2245.3) 775.2 (164.4-3126.6) 965.0 (555.8-1903.3) | 1384.4 (257.5-4168.5) 548.4 (250.0-1652.0) 1018.2 (313.4-2121.5) 1019.4 (313.5-3398.0) 1540.9 (246.4-3851.3) | 807.5 (71.0-2247.5) 872.8 (333.8-1575.1) 867.3 (330.5-2417.1) 1022.0 (346.9-4965.3) 1126.6 (441.2-2738.4) |
| IL-10 | baseline 2 hours 4 hours 6 hours 8 hours | 2.3 (1.0-5.6) 6.1 (1.7-37.8) 5.3 (1.4-13.8) 5.4 (0.6-24.4) 5.9 (1.4-16.4) | 4.2 (1.7-32.7) 8.7 (1.5-49.0) 17.6 (3.7-40.6) 16.0 (5.6-56.8) 18.4 (2.7-70.5) | 3.0 (0.4-15.9) 1.0 (1.8-42.4) 7.7 (1.8-36.8) 6.0 (1.7-31.4) 8.5 (1.1-114.1) |
| MDC | baseline 2 hours 4 hours 6 hours 8 hours | 50.9 (27.7-134.9) 69.7 (14.7-263.3) 63.4 (17.3-260.0) 49.7 (14.7-92.2) 39.9 (12.3-108.6) | 98.2 (22.5-244.9) 105.2 (10.1-245.7) 75.1 (32.3-628.4) 70.0 (33.0-427.3) 127.5 (25.6-668.5) | 68.2 (4.4-226.2) 98.3 (26.1-329.9) 86.5 (22.8-320.3) 71.2 (8.6-695.9) 109.1 (77.6-282.7) |
| RANTES | baseline 2 hours 4 hours 6 hours 8 hours | 12.5 (6.0-131.2) 13.6 (3.8-205.0) 15.3 (3.8-123.9) 7.4 (2.8-139.8) 20.1 (1.3-175.6) | 22.1 (5.6-73.5) 17.7 (5.3-94.1) 26.0 (8.5-52.7) 18.0 (6.2-72.5) 27.8 (2.9-41.0) | 12.7 (1.3-68.7) 14.8 (5.3-129.6) 11.2 (4.4-62.5) 9.4 (3.0-79.3) 17.9 (2.5-129.4) |
| ECP | baseline 2 hours 4 hours 6 hours 8 hours | 4.55 (3.2-16.3) 8.5 (4.8-13.7) 14.5 (7.0-36.9) 20.1 (4.4-25.8) 15.1 (8.9-21.2) | 10.1 (4.9-14.7) 18.7 (11.7-45.5) 40.2 (23.0-96.1) 62.5 (25.9-75.8) 77.5 (41.4-96.7) | 5.7 (3.4-14.3) 18.7 (9.4-91.3) 39.2 (25.2-134.4) 38.1 (17.7-79.2) 36.9 (20.4-81.2) |

## Slide 5
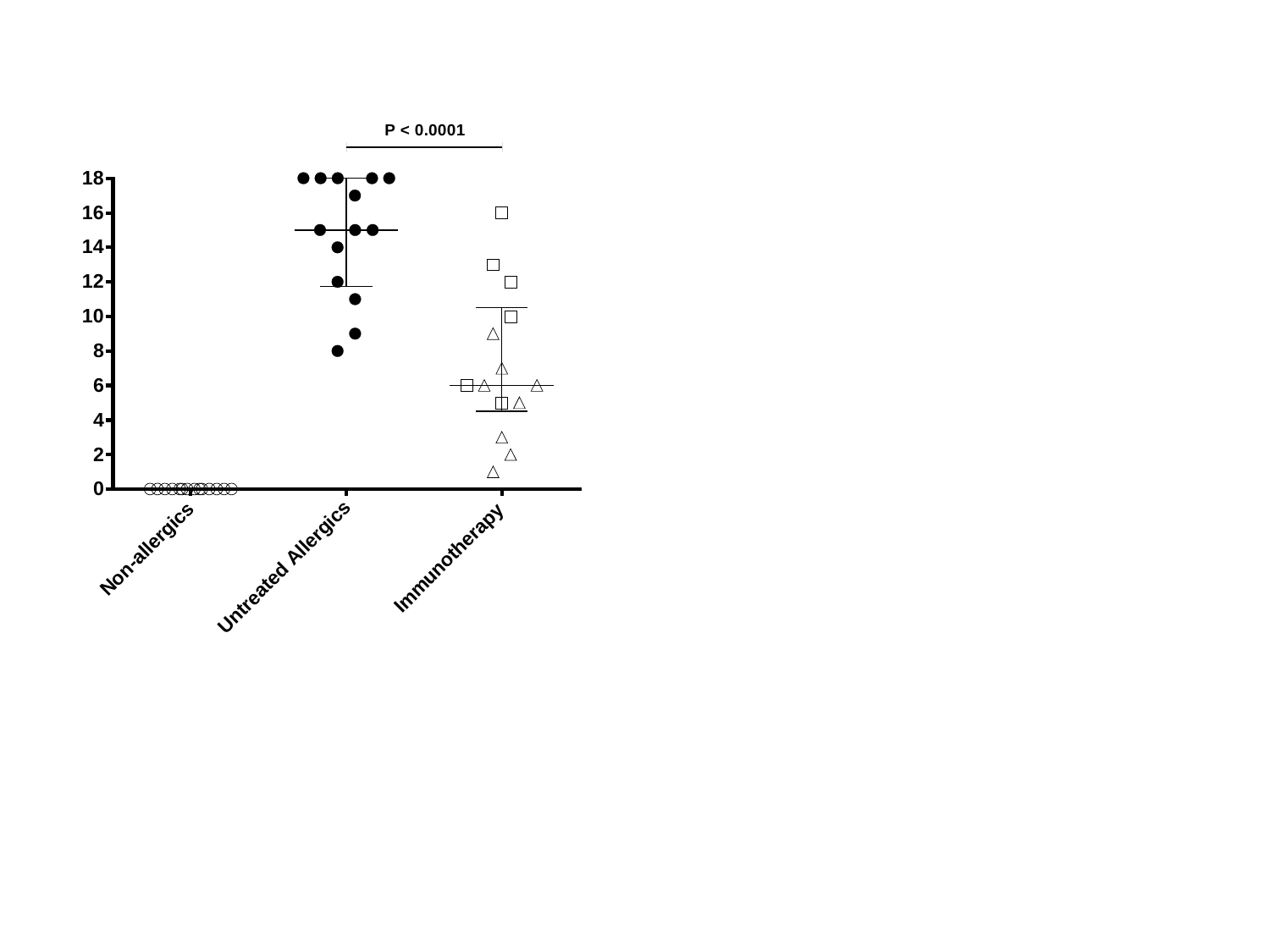

## Slide 6
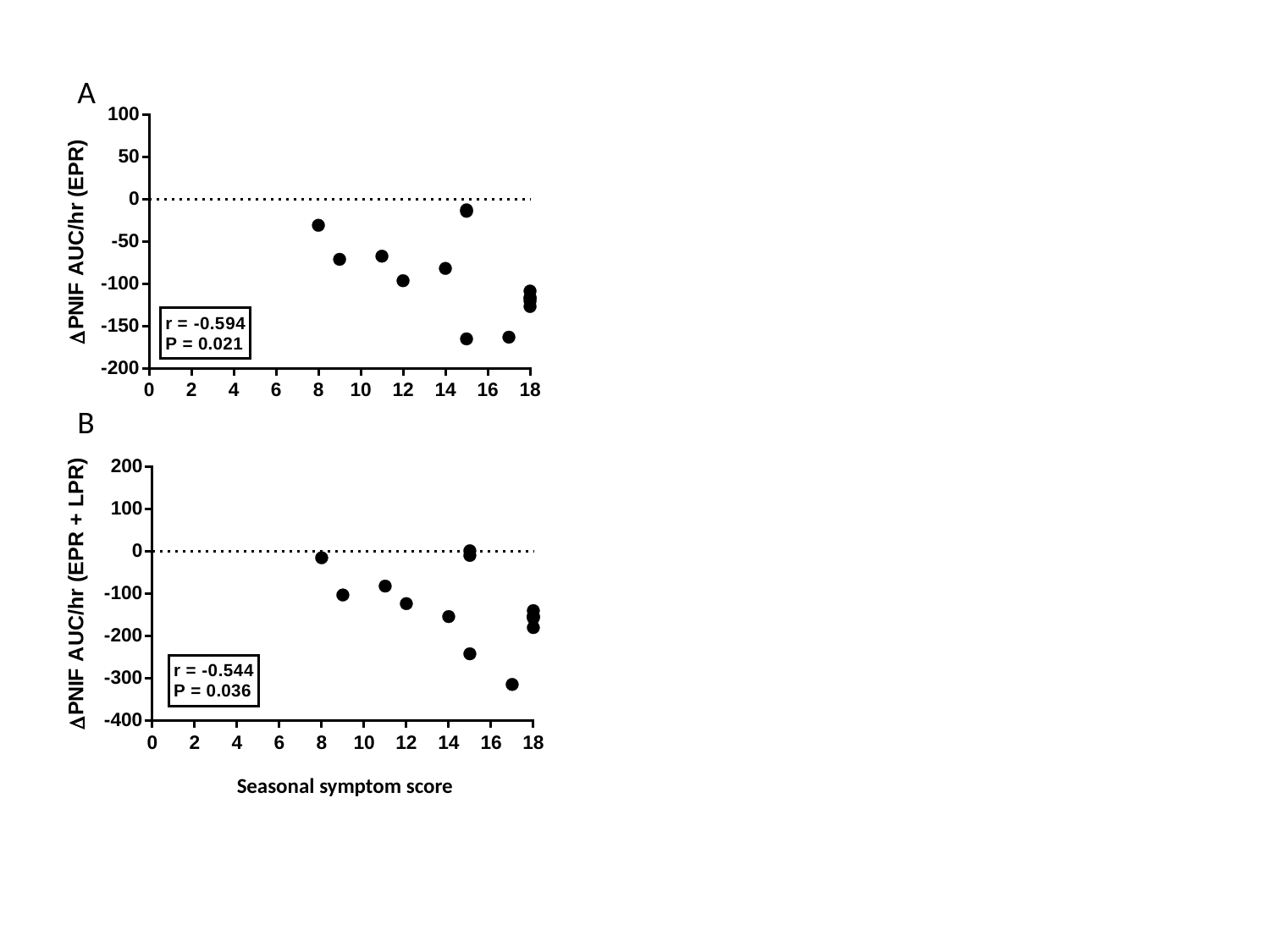

A
B
Seasonal symptom score

## Slide 7
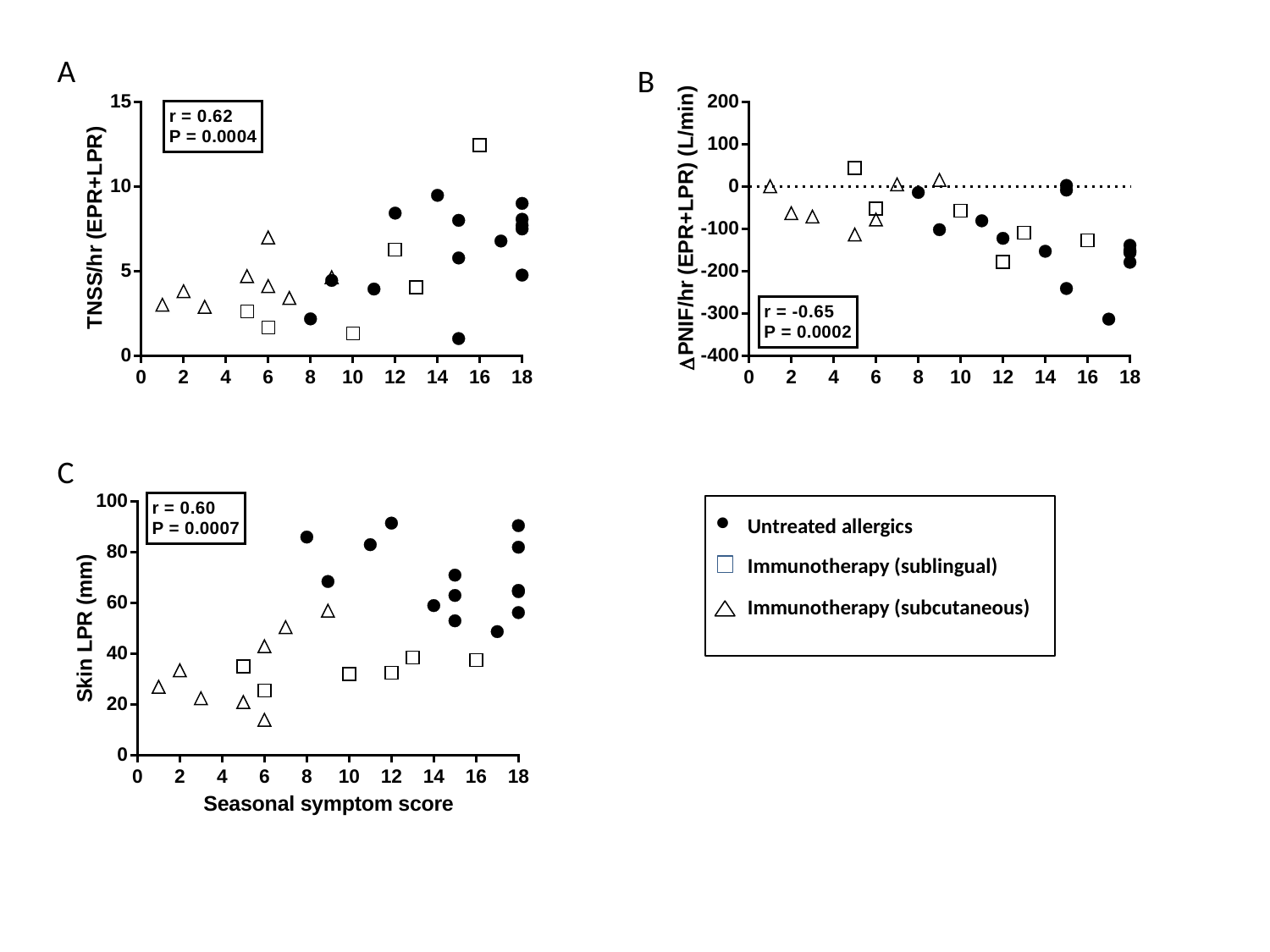

A
B
C

Untreated allergics
Immunotherapy (sublingual)
Immunotherapy (subcutaneous)
